# Brain Networks Route Neurodegeneration Patterns in Patients with Progressive Supranuclear Palsy

**DOI:** 10.1002/mds.30257

**Published:** 2025-06-09

**Authors:** Carla Palleis, Andrea Quattrone, Amir Dehsarvi, Sebastian N. Roemer‐Cassiano, Alexander M. Bernhardt, The AL‐108‐231 Investigators, the PASSPORT Study Group, Ikuko Aiba, Angelo Antonini, Diana Apetauerova, Jean‐Philippe Azulay, Ernest Balaguer Martinez, Jee Bang, Paolo Barone, Matthew Barrett, Danny Bega, Daniela Berg, Koldo Berganzo Corrales, Yvette Bordelon, Adam L Boxer, Moritz Brandt, Norbert Brueggemann, Giovanni Castelnovo, Roberto Ceravolo, Rosalind Chuang, Sun Ju Chung, Alistair Church, Jean‐Christophe Corvol, Paola Cudia, Marian Dale, Luc Defebvre, Sophie Drapier, Erika D Driver‐Dunckley, Georg Ebersbach, Karla M Eggert, Aaron Ellenbogen, Alexandre Eusebio, Andrew H Evans, Natalia Fedorova, Elizabeth Finger, Alexandra Foubert‐Samier, Boyd Ghosh, Lawrence Golbe, Francisco Grandas Perez, Murray Grossman, Deborah Hall, Kyoko Hamada, Kazuko Hasegawa, Guenter Hoeglinger, Lawrence Honig, David Houghton, Xuemei Huang, Stuart Isaacson, Jaime Kulisevsky Bojarski, Anthony E. Lang, Peter Nigel Leigh, Biomedical Research, Irene Litvan, Juan Jose Lopez Lozano, Jose Luis Lopez‐Sendon Moreno, Albert Christian Ludolph, Ma Rosario Luquin Piudo, Irene Martinez Torres, Nikolaus McFarland, Wassilios Meissner, Tiago Mestre, Pablo Mir Rivera, Eric Molho, Britt Mollenhauer, Huw R Morris, Miho Murata, Tomokazu Obi, Fabienne Ory Magne, Padraig O'Suilleabhain, Rajesh Pahwa, Alexander Pantelyat, Nicola Pavese, Dmitry Pokhabov, Johannes Prudlo, Federico Rodriguez‐Porcel, James Rowe, Joseph Savitt, Alfons Schnitzler, Joerg B Schulz, Klaus Seppi, Binit Shah, Holly Shill, David Shprecher, Maria Stamelou, Malcolm Steiger, Yuji Takahashi, Hiroshi Takigawa, Carmela Tartaglia, Lars Toenges, Kardiologische Studienambulanz, Daniel Truong, Winona Tse, Paul Tuite, Dieter Volc, Anne‐Marie A Wills, Dirk Woitalla, Tao Xie, Tatsuhiko Yuasa, Sarah Elizabeth Zauber, Theresa Zesiewicz, David Williams, Anne Louise Lafontaine, Connie Marras, Mandar Jog, Michael Panisset, Anthony Lang, Lesley Parker, Alistair J. Stewart, Jean‐Christophe Corvol, Jean‐Philippe Azulay, Philippe Couratier, Brit Mollenhauer, Stefan Lorenzl, Albert Ludolph, Reiner Benecke, Günter Höglinger, Axel Lipp, Heinz Reichmann, Dirk Woitalla, Dennis Chan, Adam Zermansky, David Burn, Andrew Lees, Adam Boxer, Bruce L. Miller, Iryna V. Lobach, Erik Roberson, Lawrence Honig, Edward Zamrini, Rajesh Pahwa, Yvette Bor‐delon, Erika Driver‐Dunkley, Stephanie Lessig, Mark Lew, Kyle Womack, Brad Boeve, Joseph Ferrara, Argyle Hillis, Daniel Kaufer, Rajeev Kumar, Tao Xie, Steven Gunzler, Theresa Zesiewicz, Praveen Dayalu, Lawrence Golbe, Murray Grossman, Joseph Jancovic, Scott McGinnis, Anthony Santiago, Paul Tuite, Stuart Isaacson, Julie Leegwater‐Kim, Irene Litvan, Murray Grossman, David S. Knopman, Bruce L. Miller, Lon S. Schneider, Rachelle S. Doody, Lawrence I. Golbe, Erik D. Roberson, Mary Koestler, Clifford R. Jack, Viviana Van Deerlin, Christopher Randolph, Iryna V. Lobach, Illana Gozes, Steve Whitaker, Joe Hirman, Michael Gold, Bruce H. Morimoto, Hans‐Jürgen Huppertz, Maura Malpetti, Adam L. Boxer, Johannes Gnörich, Lukas Frontzkowski, Johannes Levin, Matthias Brendel, Günter U. Höglinger, Nicolai Franzmeier

**Affiliations:** ^1^ Department of Neurology University Hospital, Ludwig‐Maximilians‐Universität Munich Germany; ^2^ Munich Cluster for Systems Neurology (SyNergy) Munich Germany; ^3^ German Center for Neurodegenerative Diseases (DZNE) ‐ Site Munich Munich Germany; ^4^ Neuroscience Research Centre Magna Graecia University Catanzaro Italy; ^5^ Institute of Neurology, Department of Medical and Surgical Sciences Magna Graecia University Catanzaro Italy; ^6^ Institute for Stroke and Dementia Research (ISD) University Hospital, Ludwig‐Maximilians‐Universität Munich Germany; ^7^ Max Planck School of Cognition Leipzig Germany; ^8^ Swiss Epilepsy Clinic, Klinik Lengg Zurich Switzerland; ^9^ Department of Clinical Neurosciences University of Cambridge Cambridge United Kingdom; ^10^ UK Dementia Research Institute at University of Cambridge Cambridge United Kingdom; ^11^ Department of Neurology University of California San Francisco San Francisco California USA; ^12^ Department of Nuclear Medicine University Hospital, Ludwig‐Maximilians‐Universität Munich Germany; ^13^ University of Gothenburg, The Sahlgrenska Academy Institute of Neuroscience and Physiology, Department of Psychiatry and Neurochemistry Mölndal and Gothenburg Sweden

**Keywords:** functional connectivity, gray matter atrophy, imaging, PSP, tauopathies

## Abstract

**Background:**

Progressive supranuclear palsy (PSP) is a neurodegenerative disease driven by 4‐repeat τ pathology, which is thought to propagate across interconnected neurons.

**Objectives:**

We hypothesized that interconnected brain regions exhibit correlated atrophy, and that atrophy propagates network‐like from fast‐declining epicenters to connected regions in PSP.

**Methods:**

We combined resting‐state functional magnetic resonance imaging (fMRI) connectomics with two independent 12‐month longitudinal structural magnetic resonance imaging (MRI) datasets of PSP‐Richardson syndrome (PSP‐RS) patients (*n*_discovery_/*n*_validation_ = 114/90). MRI‐based gray matter volumes were assessed for 246 regions of the Brainnetome atlas and converted to w‐scores indicating local atrophy (ie, volumes adjusted for age, sex, and intracranial volume based on regression models determined in a sample of 377 healthy amyloid‐ and τ‐negative controls from the Alzheimer's Disease Neuroimaging Initiative [ADNI]). Annual volume changes were determined for each Brainnetome region of interest using longitudinal structural MRI. Resting‐state fMRI from 69 ADNI healthy controls was used to determine a connectivity template.

**Results:**

We observed pronounced atrophy and volume decline in the frontal lobe and subcortical regions bilaterally. Correlated atrophy and volume changes were found among interconnected brain regions, with regions with severe atrophy or rapid decline being strongly connected to similarly affected areas, whereas minimally affected regions were connected to less affected areas. Connectivity patterns of atrophy epicenters predicted patient level atrophy and volume decline.

**Conclusions:**

Our findings show that key subcortical and frontal brain regions undergo atrophy in PSP‐RS and that gray matter atrophy expands across interconnected brain regions, supporting the view that neurodegeneration patterns may follow the trans‐neuronal τ propagation pattern in PSP‐RS. © 2025 The Author(s). *Movement Disorders* published by Wiley Periodicals LLC on behalf of International Parkinson and Movement Disorder Society.

Progressive supranuclear palsy (PSP) is a progressive disease driven by the accumulation of 4‐repeat (4R) τ pathology in neurons, astrocytes, and oligodendrocytes, ensuing neurodegeneration and the development of sensory‐motor and cognitive symptoms.^1^ The most common and most specific clinical manifestation of PSP‐type 4R τ pathology is Richardson's syndrome (RS), characterized by predominant oculomotor dysfunction, postural instability, and frequent falls.^1^, ^2^, ^3^ Although physiological τ is normally expressed at higher levels in cortical than in subcortical structures,^4^ postmortem studies have shown that the progression of 4R τ pathology in PSP follows a specific spatio‐temporal pattern along the pallido–nigro‐luysian axis with earliest τ aggregation in the pallidum, followed by subsequent spread to the neocortex.^5^, ^6^ This caudo‐rostral expansion has been suggested to be driven by a “prion‐like” spread of pathological τ seeds from earliest epicenters in the subcortex with subsequent propagation across synaptic connections ensuing successive template‐based τ misfolding, aggregation, and spread.^7^ This concept of trans‐synaptic τ propagation is strongly supported by previous experimental evidence, showing that hyperphosphorylated τ seeds are actively secreted from neurons at the synapse and taken up by connected neurons, and that enhanced activity in τ‐containing neurons accelerates the trans‐synaptic spread of τ both in vitro and in vivo models.^8^

We have previously translated this concept of trans‐neuronal τ spread to human PSP datasets, combining [^18^F]PI‐2620 τ‐positron emission tomography (PET), postmortem τ assessments and resting‐state functional MRI (rs‐fMRI) connectivity, showing that distribution patterns of τ pathology align closely with the connectomic architecture of the brain.^9^ Regions highly connected to each other showed similar τ pathology levels in τ‐PET assessments and in postmortem‐assessed τ levels, and connectivity patterns of epicenters with highest τ pathology were predictive of brain wide τ deposition in PSP patients.^9^ Cell‐type specific regional τ stainings revealed that neuronal τ deposits primarily drive the link between τ and connectivity, supporting synaptic connections formed by neurons as the main pathway for τ spread across brain regions. We expanded this approach to assess whether neuroinflammation and neuronal dysfunction spread with τ in PSP.^10^, ^11^ Our findings show that neuroinflammation parallels τ deposition, whereas neuronal dysfunction expands beyond sites of highest τ deposition to connected regions in a diaschisis‐like manner. Together, this suggests that the brain network acts as a general scaffold along which PSP‐type τ pathology, inflammation, and dysfunction progress.

In the current study, we aimed to determine whether neurodegeneration, that is, the assumed direct consequence of τ pathology accumulation, expands across brain networks in a similar manner. More specifically, we hypothesized that interconnected brain regions show correlated levels of gray matter atrophy and volume loss over time, and that atrophy expands from local epicenters across connected brain regions. Addressing these questions is of key clinical relevance, since predicting neurodegenerative trajectories in PSP, ideally on the patient level, could aid with patient‐centered disease prognostication, and the determination of patient‐specific readouts of neurodegeneration in disease‐modifying trials. To this end, we leveraged longitudinal structural MRI data of two independent cohorts of patients with PSP‐RS from existing placebo arms of large‐scale clinical trials.^12^, ^13^ We first assessed regional patterns of gray matter atrophy using an age‐matched sample of healthy control subjects as a reference as well as annual gray matter volume loss using longitudinal structural MRI data. To model the spread of atrophy across interconnected brain regions, we leveraged high‐resolution fMRI data from healthy controls of the Alzheimer's Disease Neuroimaging Initiative (ADNI) cohort to determine a functional connectivity template, used as a scaffold for the inter‐regional expansion of neurodegeneration.^9^ Combining structural MRI data from the PSP cohorts with the rs‐fMRI template, we tested (1) whether connected brain regions show correlated brain atrophy (ie, cross‐sectional) and correlated volume loss over time (ie, longitudinal); (2) whether atrophy and volume loss propagate from local epicenters across connected regions; and (3) whether integrating brain atrophy patterns and connectivity can predict brain atrophy and progressive volume loss on the patient level.

## Patients and Methods

### Sample

For our primary analyses, we included two independent multisite cohorts of patients with PSP‐RS from the placebo arms of randomized controlled trials (PASSPORT [NCT03068468],^12^ and AL‐108‐231 Davunetide [NCT01110720]^13^). Detailed information and inclusion criteria can be found online (https://clinicaltrials.gov) with the respective trial identifiers and in a previous publication.^14^

As a healthy reference cohort (HC) to determine regional brain atrophy patterns in the PSP‐RS cohorts, we further included 3 Tesla (T) structural MRI data from individuals from the ADNI (ClinicalTrials.gov ID: NCT02854033). For assessing brain connectivity, we included rs‐fMRI data from a selected subset of ADNI subjects.

Ethical approval for each study (ie, PASSPORT, AL‐108‐231 and ADNI) was obtained at each site from the local ethics committee, and all participants gave written informed consent in accordance with the Declaration of Helsinki. Details on the cohorts, study protocols, structural MRI acquisition and processing; and assessment of a functional connectivity template are listed in the Supporting Data.

### Statistics

All analyses described here were conducted using R statistical software (R version 4.3.1). Associations (standardized β‐weights and correlations) were considered significant when meeting an α threshold of 0.05. Sample demographics were compared between the groups using *t* tests for continuous measures and χ^2^ tests for categorical measures.

For our first aim, we evaluated the relationship between gray matter atrophy, longitudinal volume changes, and inter‐regional functional connectivity. To test whether connected brain regions exhibit correlated atrophy or volume changes, linear regression models were applied, using inter‐regional connectivity‐based distance as a predictor of covariance in gray matter w‐scores and longitudinal volume change rates. Analyses were conducted separately for the discovery and validation cohorts to ensure reproducibility and assessment of robustness.

For our second aim, we determined whether neurodegeneration spreads from circumscribed epicenter regions across connected regions, using seed‐based regression models. Specifically, regions of interest (ROIs) were rank‐ordered by their baseline gray matter atrophy (ie, w‐scores) or longitudinal volume change rates within each patient group. For each rank‐ordered ROI, seed‐based connectivity from the ADNI template to all other ROIs was used as a predictor of their respective group‐mean atrophy or volume change levels in other ROIs using linear regression.

Finally, to address aim three, patient‐level models were implemented to evaluate whether baseline and longitudinal gray matter changes could be predicted by the connectivity profile of epicenter regions with highest baseline atrophy or fastest volume decline. For each patient, regions with the strongest atrophy or fastest volume loss were identified, and their connectivity‐based distances to other ROIs were computed using the ADNI connectivity template. Linear regression models were used to determine the relationship between these distances and gray matter atrophy or volume loss in the remaining ROIs. Mean standardized regression coefficients were calculated to summarize the results across the cohort, with confidence intervals (CI) and statistical significance tested using one‐sample *t* tests against zero.

## Results

The final study cohort included 204 patients with PSP‐RS with longitudinal clinical and 3 T MRI assessment, split into a discovery cohort (Passport, *n*_discovery_ = 114; 64 [56%] male; mean [SD] age 69.7 [5.7] years) and an independent validation cohort (AL‐108‐231 Davunetide, *n*_validation_ = 90; 42 [47%] male; mean [SD] age 67.3 [6.9] years). Group demographics are displayed in Table 1. Gray matter volume was computed for comparison in 377 age‐ and sex‐matched HC (154 [46%] male; mean [SD] age 72.6 [6.5] years) from the ADNI cohort. All results were fully replicated across both samples.

**TABLE 1:** Group demographics

	Discovery cohort: Passport study	Validation cohort: AL‐108–231 study	*P*‐value
n	114	90	
n longitudinal	114	89	
Sex (male in %)	64 (56)	42 (47)	0.23
Age in y (SD)	69.65 (5.69)*	67.32 (6.86)*	0.01
PSPRS total score (SD)	36.74 (10.11)	38.23 (10.68)	0.31

Age and PSPRS distribution were compared with *t* test; sex distribution was compared with χ^2^ test. Results are displayed as mean (SD).

Abbreviations: SD, standard deviation; PSPRS, PSP‐rating scale.

^*^ *P* < 0.05.

### Connected Brain Regions Show Correlated Gray Matter Atrophy and Volume Changes

**FIG. 1:**
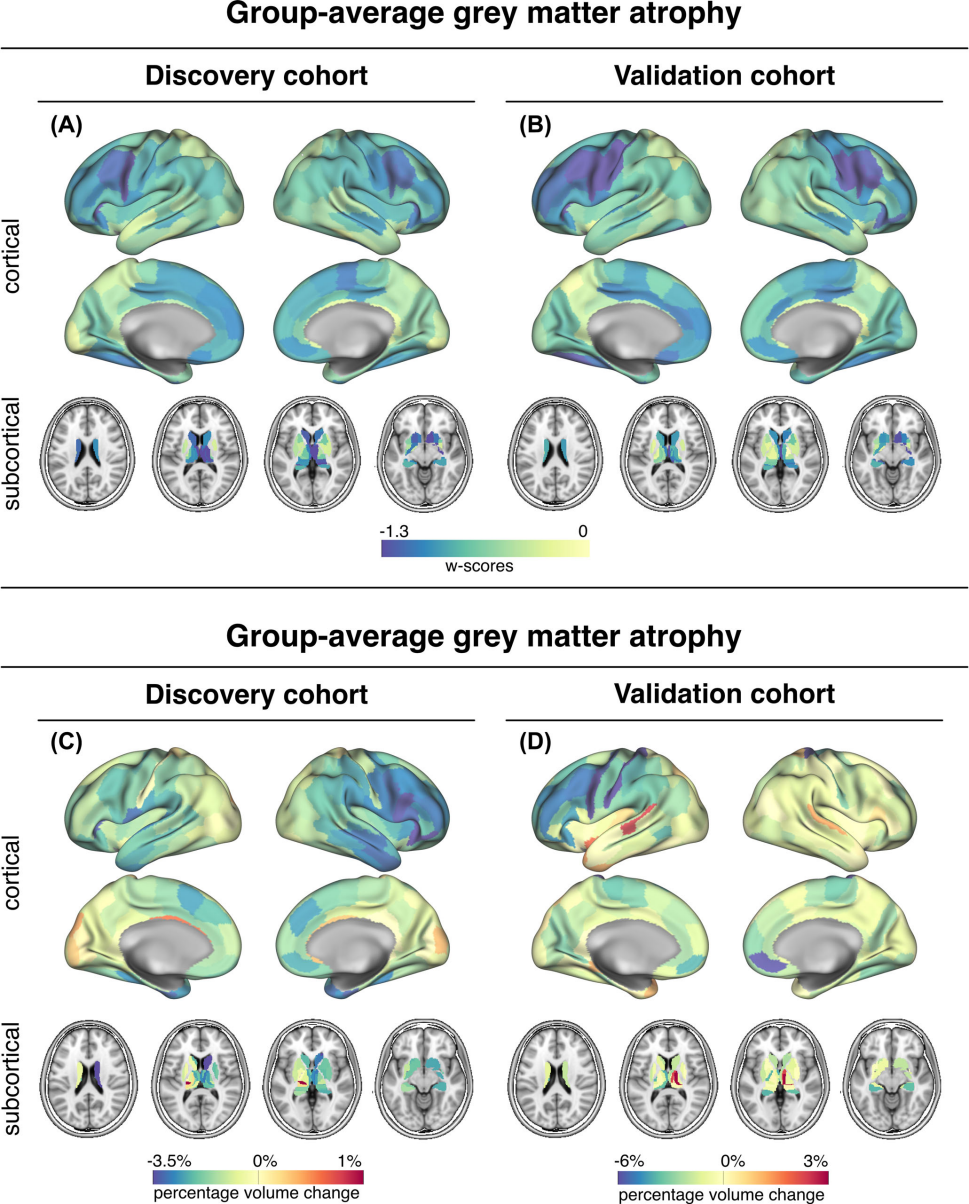
Group‐average cross‐sectional (A&B) and longitudinal gray matter atrophy (C&D). Mean distribution of cross‐sectionally‐assessed gray matter atrophy was measured with w‐scores (lower scores = stronger volume reduction) for each sample and diagnostic group. [Color figure can be viewed at wileyonlinelibrary.com]

For our first aim, we assessed whether interconnected brain regions show correlated brain atrophy and volume changes over time. Mapping brain atrophy patterns, we found strongest baseline volume reductions (ie, lower w‐scores) in subcortical and frontal brain regions, consistently across the discovery (Fig. 1A) and validation cohort (Fig. 1B). Similarly, fastest rates of gray matter volume loss over time were found mostly in subcortical and fronto‐temporal brain regions in the discovery cohort and in left frontal brain regions in the validation cohort (Fig. 1C,D).

**FIG. 2:**
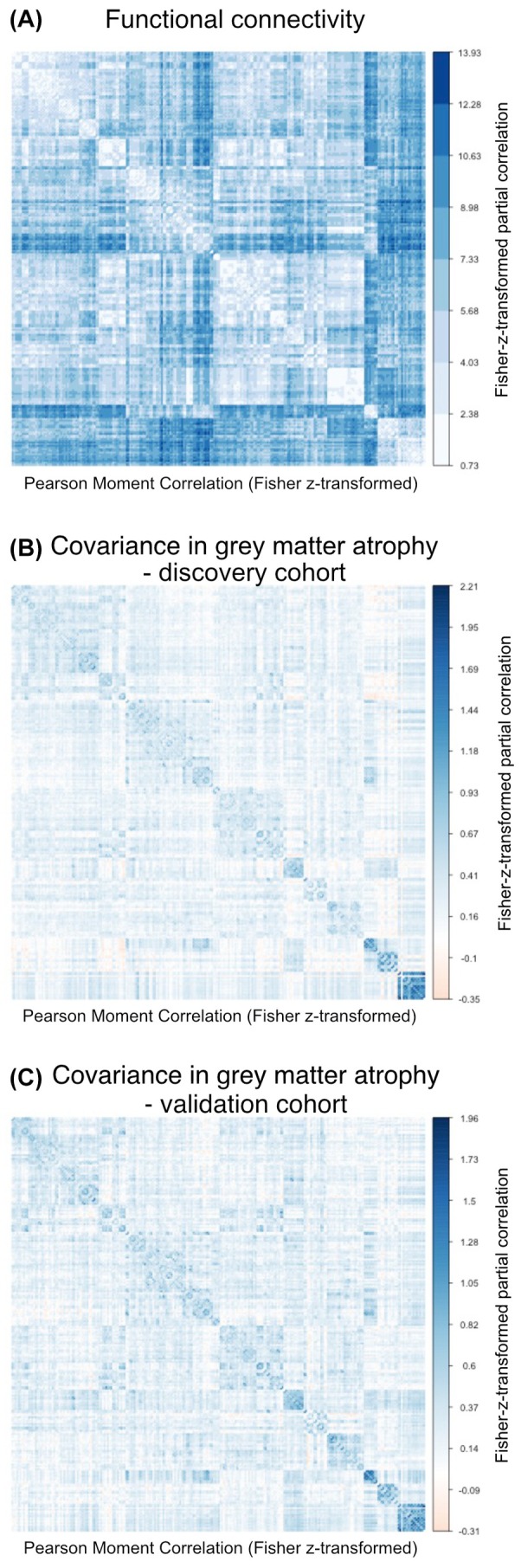
Group‐average functional connectivity and covariance in gray matter atrophy matrices. Group‐average functional connectivity was computed based on resting‐state functional magnetic resonance imaging (fMRI) of 69 cognitively normal, amyloid‐PET and τ‐PET negative Alzheimer's Disease Neuroimaging Initiative (ADNI) participants (**A**). The covariance matrices are shown for the discovery cohort (**B**) and for the validation cohort (**C**). [Color figure can be viewed at wileyonlinelibrary.com]

**FIG. 3:**
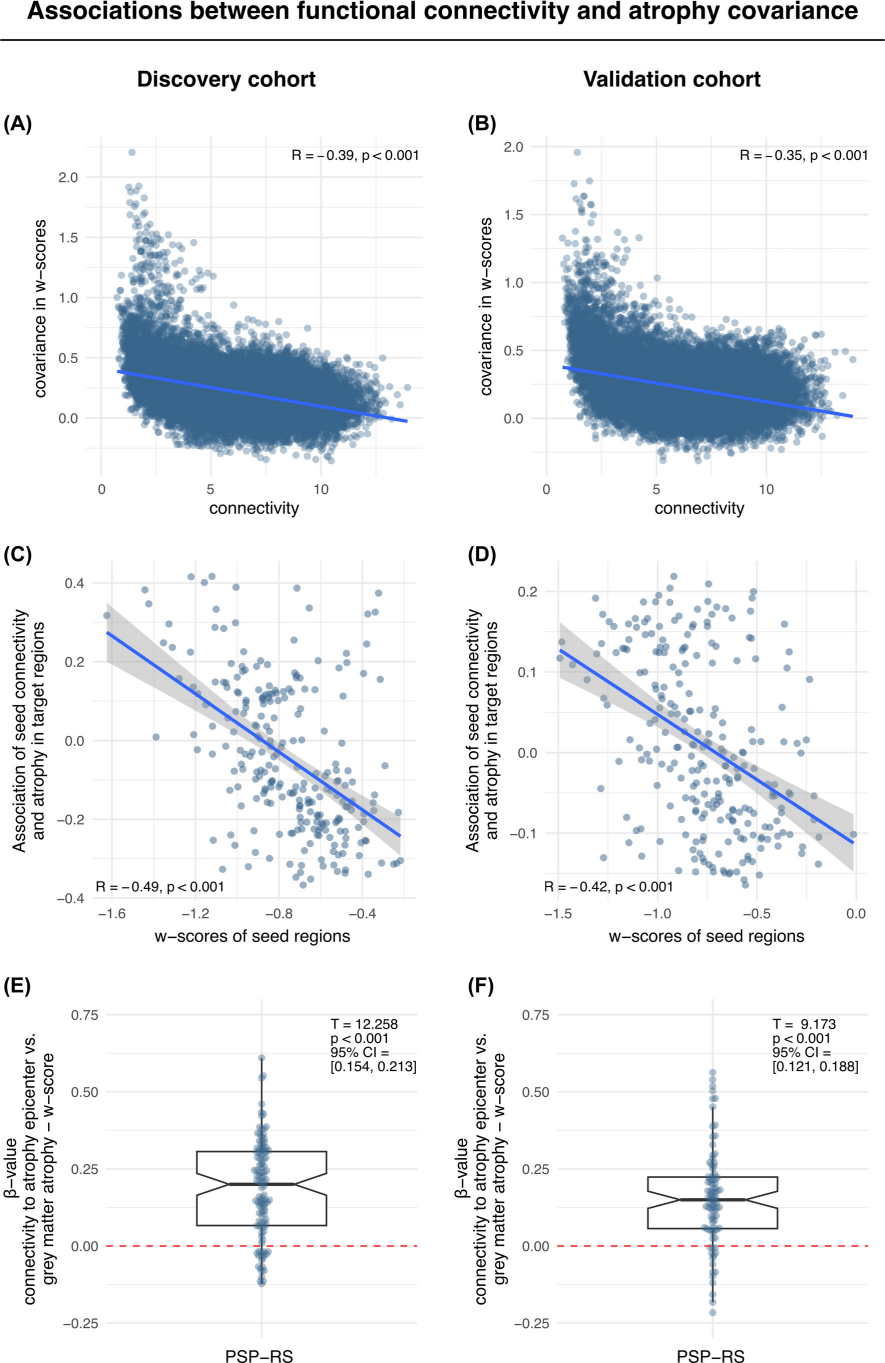
Associations between functional connectivity and atrophy covariance. The association between functional connectivity and covariance in w‐scores is illustrated in the scatter plot for the discovery cohort (**A**) and for the validation cohort (**B**). Note that connectivity is displayed as connectivity distance, where shorter distance indicates stronger connectivity. ROI‐based analyses between seed‐based connectivity and w‐scores were performed on subject‐level data, yielding a regression‐derived β‐value for the association between a given seed ROIs connectivity and w‐scores in the discovery (**C**) and validation cohort (**D**), showing that connectivity of regions with low w‐scores (ie strong atrophy) predicts brain wide atrophy patterns. The distribution of subject‐level β‐values between gray matter atrophy epicenter connectivity and w‐scores is shown in the box plots for the discovery cohort (**E**) and the validation cohort (**F**), respectively, illustrating that regions more strongly connected to a particular patients gray matter atrophy epicenter also show stronger atrophy. CI, confidence interval. [Color figure can be viewed at wileyonlinelibrary.com]

Assessing whether connected brain regions show correlated brain atrophy, we found a significant association between shorter inter‐regional connectivity‐based distance (ie, shorter distance = stronger connectivity) (Fig. 2A) and stronger inter‐regional covariance in gray‐matter w‐scores (Fig. 2B,C). This was consistent across the discovery cohort of 114 patients with PSP‐RS (β = −0.39, *P* < 0.001) (Fig. 3A) and the validation cohort of 90 patients with PSP‐RS (β = −0.35, *P* < 0.001) (Fig. 3B).

**FIG. 4:**
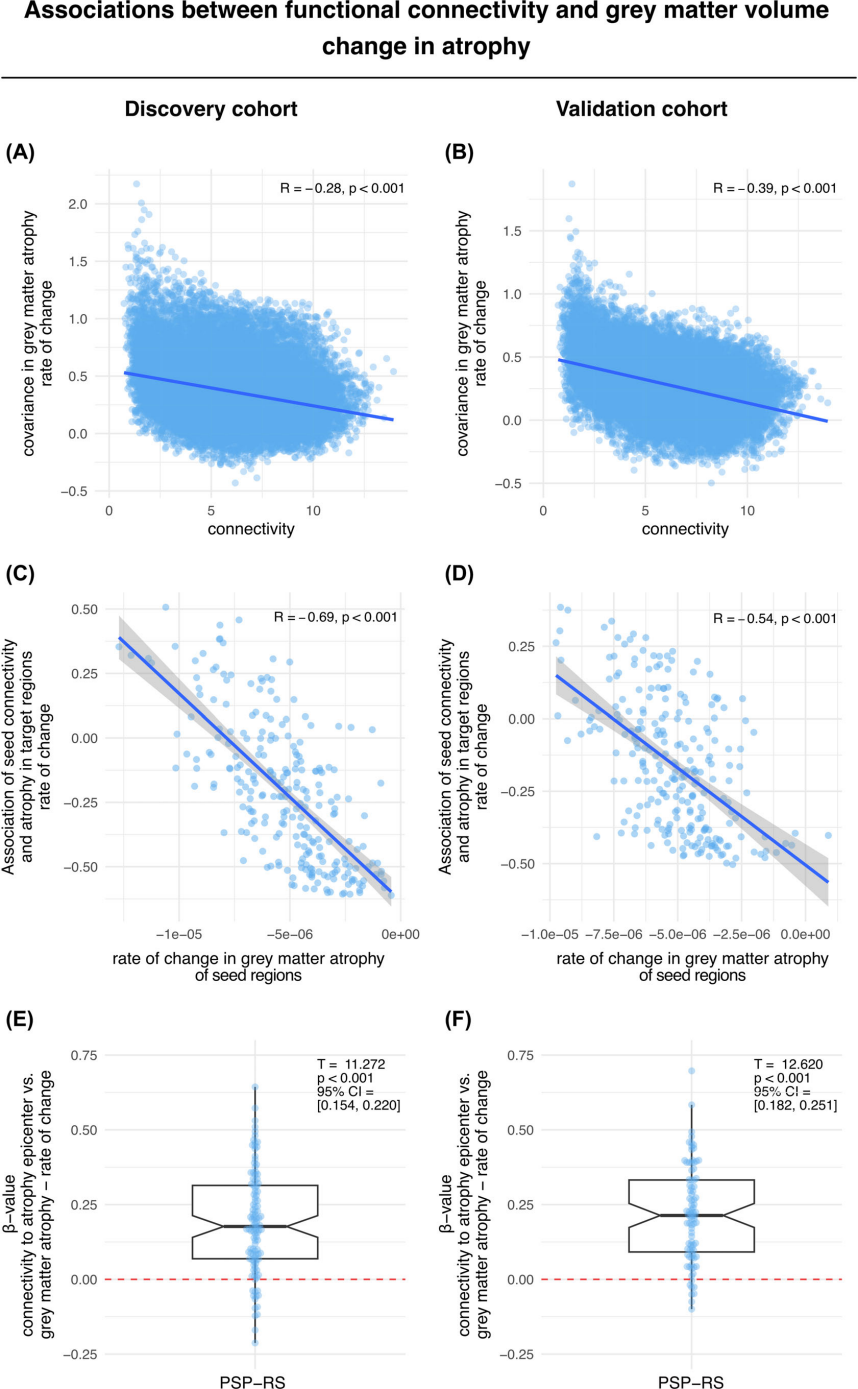
Associations between functional connectivity and gray matter volume change in atrophy. The association between functional connectivity and rate of change of gray matter atrophy is illustrated in the scatter plot for the discovery cohort (**A**) and for the validation cohort (**B**). Note that connectivity is displayed as connectivity distance, where shorter distance indicates stronger connectivity. ROI‐based analyses between seed‐based connectivity and gray matter atrophy rates were performed on subject‐level data, yielding a regression‐derived β‐value for the association between a given seed ROIs connectivity and gray matter volume change in the discovery (**C**) and validation cohort (**D**), showing that connectivity of regions with fastest volume decline predicts brain wide volume decline. The distribution of subject‐level β‐values between gray matter volume decline epicenter connectivity and brain wide volume decline is shown in the box plots for the discovery cohort (**E**) and the validation cohort (**F**), respectively, illustrating that regions more strongly connected to a particular patients epicenter also show stronger volume decline. CI, confidence interval. [Color figure can be viewed at wileyonlinelibrary.com]

When applying the same analysis approach to longitudinally assessed gray matter change rates, we could confirm that more strongly connected brain regions also show correlated gray matter volume loss over time consistent across the discovery (β = −0.28, *P* < 0.001) (Fig. 4A) and validation sample (β = −0.39, *P* < 0.001) (Fig. 4B).

To address aim two whether neurodegeneration spreads from circumscribed epicenters across connected brain regions, we extended the previous analysis by testing whether the level of baseline gray matter atrophy or longitudinal volume change in a given seed ROI is associated with the level of baseline gray matter atrophy or longitudinal volume change in closely connected regions. This analysis is based on the idea that if neurodegeneration spreads from local epicenters as a function of functional connectivity, then the connectivity of regions with strong atrophy or fast volume change should be predictive of atrophy or volume change in the rest of the brain. To test this hypothesis, we rank‐ordered all ROIs according to their level of gray matter atrophy or longitudinal volume changes, respectively. We reasoned that at strong levels of gray matter atrophy or fast volume loss in seed regions, stronger connectivity should be associated with stronger atrophy or faster volume loss in target regions. Vice versa, at lower levels of gray matter atrophy of slow volume loss over time in the seed regions, stronger connectivity should be associated with less atrophy and slower volume loss. Using linear regression, we tested for each rank‐ordered ROI (seed) within each sample, the group‐average connectivity to the remaining ROIs (target) as a predictor of the group‐average level of gray matter atrophy or volume loss over time. As hypothesized, we consistently found in both samples that depending on the level of gray matter atrophy in the seed region, the predictive value of connectivity for the level of gray matter atrophy in the target region changed. Specifically, for seed ROIs with strong gray matter atrophy at baseline (ie, more negative w‐scores), stronger connectivity (ie, shorter connectivity‐based distance) was associated with stronger gray matter atrophy in target regions (ie, positive β‐values in the regression). Conversely, for seeds with low gray matter atrophy at baseline (ie, more positive w‐scores), stronger connectivity was associated with lower gray matter atrophy in target regions (ie, negative β‐values in the regression). This result pattern was mirrored in a strong negative association between the seed ROIs' gray matter atrophy and their connectivity's predictive weight (ie, regression derived β‐value) on gray matter atrophy in target ROIs in both the discovery (β = −0.49, *P* < 0.001) (Fig. 3C) and validation sample (β = −0.42, *P* < 0.001) (Fig. 3D).

Applying this analysis to longitudinally assessed change rates yielded a consistent result pattern, where regions with faster volume changes were connected preferentially to other regions with fast volume changes in the discovery (β = −0.69, *P* < 0.001) (Fig. 4C) and validation sample (β = −0.54, *P* < 0.001) (Fig. 4D). Together, these findings suggest that interconnected brain regions show correlated gray matter atrophy and volume loss over time in patients with PSP‐RS, consisting with the hypothesis that atrophy spreads from local epicenters across connected brain regions.

### Patient‐Level Prediction of Gray Matter Atrophy and Longitudinal Volume Changes

For aim three, we tested whether subject level patterns of baseline gray matter atrophy or longitudinal volume change can be predicted based on the seed‐based connectivity of those epicenter regions with highest baseline atrophy or fastest longitudinal volume decline. For baseline gray matter atrophy, we determined atrophy epicenters for each patient defined as 10% of brain regions with the lowest w‐scores.^10^ For longitudinal data, epicenters were defined for each patient as 10% of brain regions with fastest volume decline. For each patient baseline and longitudinally defined epicenters, we then extracted the mean seed‐based functional connectivity‐based distance to the remaining ROIs using the ADNI‐derived connectivity template. We, then, applied subject‐specific linear regression models to assess whether the shorter connectivity‐based distance of the baseline atrophy or longitudinal volume change epicenters to other regions predicted stronger atrophy or faster volume decline. For baseline atrophy, the mean standardized β‐coefficients of the association between epicenter connectivity and baseline gray matter atrophy were significantly greater than zero both in the discovery sample (β_mean_ = 0.184, 95% CI = [0.154–0.213], *T* = 12.258; *P* < 0.001) (Fig. 3E) as well as the validation sample (β_mean_ = 0.155, 95% CI = [0.121–0.188], *T* = 9.173, *P* < 0.001) (Fig. 3F). Similarly, for longitudinal gray matter change, the mean standardized β‐coefficients of the association between epicenter connectivity and longitudinal volume change were significantly greater than zero in both the discovery (β_mean_ = 0.187, 95% CI = [0.154–0.220], *T* = 11.272, *P* < 0.001) (Fig. 4E) and validation sample (β_mean_ = 0.216, 95% CI = [0.182–0.251], *T* = 12.620, *P* < 0.001) (Fig. 4F). Clinical decline (ie, change in the longitudinal scores of the PSP‐rating scale [PSPRS]) was associated with subject‐level epicenter atrophy rates (ie, regions with fastest volume decline over time) in the discovery cohort, but not in the validation cohort (Supporting Figure S1).

These findings indicate that subject‐level gray matter atrophy patterns and longitudinal change rates follow the connectivity pattern of epicenters with highest atrophy and fastest volume decline, supporting the view that the brains connectomic architecture acts as a scaffold for routing atrophy spread on the patient level, but this volume decline does not necessarily translate into stronger clinical decline.

## Discussion

The main goal of this study was to assess whether neurodegeneration in PSP patients—believed to result directly from the accumulation and spread of τ pathology^15^—progresses across interconnected brain regions, using the brains' connectomic architecture as a general scaffold. Specifically, we hypothesized that functionally connected brain regions would exhibit similar patterns of gray matter atrophy and volume loss over time, with atrophy spreading from localized epicenters to adjacent connected regions. To this end, we assessed T_1_ MRI‐based gray matter volumes and volume change over time of two independent large cohorts of patients with clinical diagnosis of probable PSP‐RS.^12^, ^13^ Rs‐fMRI from 69 ADNI healthy controls^9^ was used to determine a connectivity template across which we modelled spread of gray matter atrophy. Strongest gray matter atrophy and longitudinal volume loss were found bilaterally in the frontal lobe and in basal ganglia as expected from MRI imaging^14^, ^16^, ^17^ and histopathological studies.^5^, ^6^ Further, the atrophy patterns closely align with regions of elevated [^18^F]PI‐2620 τ‐PET binding in PSP patients, primarily involving the basal ganglia and frontal lobes.^9^, ^18^, ^19^ We found that higher functional connectivity was associated with stronger covariance of gray matter atrophy as well as correlated volume change over time. Regions with severe atrophy or rapid volume loss were strongly connected to similarly affected regions. Moreover, the seed‐based connectivity patterns of epicenters with highest baseline atrophy or fastest volume change predicted brain‐wide atrophy and volume change patterns on the subject level. All results were fully replicated across both cohorts.

Our first main finding shows that levels of atrophy are similar (ie, covary) across regions that are highly functionally connected, which is consistent with a previous study investigating cross‐sectional functional MRI in a cohort of 36 patients with PSP‐RS^20^ showing that network connectivity of subcortical atrophy epicenters predicts downstream atrophy in connected regions. Similarly, by combining [^18^F]PI‐2620 τ‐PET and rs‐fMRI, we have shown previously that regions that are highly connected to each other show similar τ levels in 4R tauopathy patients,^9^ where this association was mainly driven by neuronal τ. Further, [^18^F]PI‐2620 τ deposition in subcortical predilection sites may induce cortical neuronal dysfunction in form of cerebral hypoperfusion in connected regions, that may precede macrostructural neurodegeneration detectable with MRI.^10^ Therefore, these findings are in line with the concept that τ spreads from local epicenters across interconnected neurons that form the basis of neuronal networks and that the patterns of τ‐induced neuronal dysfunction and neurodegeneration follow the trans‐neuronal τ propagation in PSP along connected brain regions.^15^ This is also suggested for other tauopathies, such as the 3/4‐repeat tauopathy Alzheimer's disease (AD), where highly interconnected regions determined on rs‐fMRI are associated with higher spatial covariance of τ deposition and gray matter atrophy.^21^, ^22^, ^23^, ^24^, ^25^ The concept of trans‐synaptic τ propagation is strongly supported by experimental evidence demonstrating that hyperphosphorylated τ seeds are actively secreted and taken up by connected neurons at synapses, and that increased activity in τ‐containing neurons accelerates trans‐synaptic τ spread in both in vitro and in vivo models.^8^, ^26^, ^27^ Further, in the optogenetically stimulated τ transgenic mouse model an increased spread of τ is followed by exacerbated cell atrophy.^8^ Although physiological τ can be expressed at higher levels in cortical neurons than in subcortical structures,^4^ postmortem staging studies on PSP patients suggest that τ deposits appear early in the disease course, sequentially spreading from midbrain and pons and ultimately to neocortex, accompanied by significant neuronal loss and the development of clinical symptoms.^5^, ^28^ Therefore, the pattern of atrophy spread we observed along interconnected regions likely reflects a combination of intrinsic regional vulnerability and trans‐neuronal spread of pathological τ rather than a direct relationship to normal τ expression levels. As the disease progresses, atrophy becomes increasingly pronounced as measured with structural MRI,^14^, ^29^ aligning with the postmortem staging system of progressive τ aggregation.^5^ Atrophy has been shown to be linked to worsening of symptoms^16^, ^30^ and subcortical atrophy may serve as a predictor of survival in PSP.^17^ However, regarding the association between clinical worsening in the PSPRS and epicenter atrophy rate, we found inconsistent results in the two large cohorts in our study, although both cohorts had a similar atrophy pattern and volume loss over time. This emphasizes that brain atrophy may not relate uniformly to the progression of clinical disease severity, as also outlined in our previous study.^14^ In addition, epicenters encompass different brain regions per individual, hence their atrophy may not uniformly relate to the symptoms captured in the PSPRS score.

There is increasing evidence that the colocalization of neuroinflammation with τ aggregation may be a further factor contributing to disease progression and atrophy in tauopathies like AD,^31^, ^32^ and PSP.^33^, ^34^ We have shown recently that neuroinflammation may even precede the spread of τ pathology across connected regions as measured with τ‐ and translocator protein (TSPO)‐PET.^11^ Inflammatory factors appear capable of triggering neuronal τ aggregation, promoting τ spreading, and driving τ‐induced synaptic dysfunction.^35^, ^36^ This is supported by experimental evidence that in P301S τ transgenic mice microglial activation may occur before the formation of τ tangles early in the disease course.^37^ Taken together, brain atrophy in tauopathies results from a complex interplay between protein aggregation and neuroinflammation that spreads across connected brain regions, with each process exacerbating the other and contributing to neuronal loss and tissue shrinkage across connected regions.

To further support the hypothesis that gray matter atrophy progresses across brain networks in PSP, we investigated if functionally connected brain regions would exhibit similar patterns of gray matter volume loss over time. We found that regions with fast volume decline were strongly connected to other fast declining regions, similar to gray matter loss following a specific progression pattern in AD.^38^, ^39^ In line with this, transgenic mouse model studies investigating tauopathy spread have shown a strong association between the propagation of τ pathology and volume loss in longitudinal structural MRI^37^, ^40^ and fMRI,^41^, ^42^ suggesting that τ aggregation interferes with normal cellular processes, including microtubule stability and axonal transport, which lead to cellular dysfunction and subsequent brain atrophy across connected regions following the spread of τ. This aligns with previous findings that, although loss of normal τ function can cause axonal instability in τ knockout mice,^43^ overall τ loss of function alone plays a limited role of neuronal degeneration in τ related diseases.^44^, ^45^

Taken together, the spread of τ in PSP may be approximated by examining the patterns of atrophy in brain regions that are functionally connected. Given that τ pathology likely drives neurodegeneration in PSP through toxic gain‐of‐function mechanisms, mapping gray matter loss across functionally linked regions may provide valuable insights into the pathways of τ propagation. By tracking the volume changes in connected brain areas, clinical research could use this information as a “surrogate endpoint” in clinical trials of PSP‐RS patients. Instead of waiting to observe long‐term clinical outcomes (like changes in movement or cognition), measurable atrophy over time along connected brain regions may be used as indicators of the disease's progression and the potential effectiveness of treatments. Although this method does not currently aim to replace established imaging markers such as midbrain atrophy, it may complement them by providing a network‐level perspective on brain atrophy. The advantage of a MRI biomarker as a surrogate endpoint is its wide availability, making it accessible for use in diverse clinical and research settings. However, further methodological refinement and validation are needed before this approach can be applied to power calculations or sample size estimations in interventional studies.

There are some limitations to the study. First, there is no histopathological confirmation of the diagnosis. To reduce potential misdiagnosis, the screening of PSP‐RS patients was done by movement disorders specialists in both trials, and only patients with diagnosis of a probable PSP pathology^1^ were included in our analysis. Further, the discrepancy between clinical and histopathological diagnoses in PSP‐RS phenotype is generally low.^3^ We acknowledge that the participants were moderately affected at baseline (PSPRS ~35), limiting conclusions about early‐stage disease. Because this study involved two distinct multi‐site cohorts, a potential further limitation could be the technical variability of different scanners used across sites, even though MRI acquisition protocols were matched. Nevertheless, the similarity in atrophy patterns observed in both groups, despite these protocol differences, suggests that the discrepancies did not significantly affect the reliability of our findings, endorsing the applicability as a possible biomarker in real‐world clinical settings.

In addition, as there are not yet sufficiently large, good quality rs‐fMRI data in the investigated PSP cohorts, the template of connectivity across brain regions was determined on rs‐fMRI data from healthy controls, although brain connectivity patterns may differ between patients with PSP and normally aged elderly.^46^ Changes in connectivity may influence spreading of pathology and atrophy, which should be specifically addressed in future studies, investigating the role of brain connectivity changes in routing τ and brain atrophy spread and ultimately disease progression. Additionally, 3 T rs‐fMRI faces significant challenges when applied to the brainstem because of its small and complex structure of nuclei and fiber tracts, making it challenging to spatially resolve and interpret functional activity. Our results are based on conventional 3 T structural MRI data from large‐scale clinical cohorts, where sequences were not tailored to specifically delineate brainstem nuclei and their volume. The role of connectivity between brainstem and subcortical and cortical regions in PSP should be specifically addressed by dedicated studies, using high‐resolution and target imaging of relevant brainstem structures. Therefore, conclusions about spread of network disruptions in early PSP disease are limited in the current study. Further, future work should be extended to PSP phenotypes other than PSP‐RS, which is the enrolled phenotype in the two trials assessed in this study, as the clinical variety of the PSP phenotypes are thought to be related to differences in the propagation patterns of τ pathology.^5^, ^47^, ^48^ Last, we did not combine gray matter volume assessments with [^18^F]PI‐2620 τ‐PET to simultaneously track 4R τ aggregation and neurodegeneration, as PET scanning was not implemented in the clinical trial protocols. Therefore, it remains open whether the neurodegeneration patterns are directly related to 4R τ deposition, although previous studies have provided compelling evidence that 4R τ triggers neurodegeneration and brain atrophy.^5^, ^15^ Future studies should combine both PET and structural MRI to further elucidate the pathophysiological trajectories and timing of τ accumulation and neurodegeneration in PSP.

In conclusion, our findings suggest that in PSP patients, interconnected brain regions demonstrate correlated patterns of gray matter atrophy and volume loss over time, with the progression of atrophy originating from localized epicenters and spreading across functionally connected areas. Together with previous evidence, this is supporting the view that gray matter atrophy may follow trans‐neuronal τ propagation in PSP. This is of substantial clinical importance, because understanding the spatial and temporal dynamics of neurodegeneration in PSP could enhance our ability to predict disease trajectories, particularly at the level of individual patients. This may be useful for treatment strategies tailored to the unique progression patterns in each patient. Furthermore, our findings underscore the potential of identifying patient‐specific biomarkers of neurodegeneration, which could serve as relevant endpoints in clinical trials aimed at modifying the course of PSP.

## Author Roles

C.P.: Execution, visualization, analysis, writing, editing of final version of the manuscript. A.Q.: Execution, analysis, editing of final version of the manuscript. A.D.: Execution, analysis, editing of final version of the manuscript. S.R.‐C.: Resources, editing of final version of the manuscript. A.M.B: Resources, editing of final version of the manuscript. H.‐J.H.: Execution, analysis, editing of final version of the manuscript. M.M.: Resources, editing of final version of the manuscript. A.L.B.: Resources, editing of final version of the manuscript. J.G.: Resources, editing of final version of the manuscript. L.F.: Resources, editing of final version of the manuscript. J.L.: Resources, editing of the final version of the manuscript. M.B.: Design, resources, editing of the final version of the manuscript. G.U.H.: Conceptualization, data acquisition, design, writing, editing of the final version of the manuscript. N.F.: Conceptualization, execution, visualization, analysis, writing, editing of final version of the manuscript.

## Supporting information


**Figure S1.** Association between epicenter atrophy rate and PSPRS change rate.

**Data S1.** Supporting Information.

## Data Availability

The data were collected within the context of clinical trials or observational studies and can be requested from the respective owners of the data.

## A.1 Passport study group

Ikuko Aiba (National Hospital Organization Higashinagoya National Hospital, Nagoya, Aichi, Japan), Angelo Antonini (San Camillo Hospital IRCCS, Venice Lido, Italy), Diana Apetauerova (Lahey Hospital and Medical Center, Burlington, MA, USA), Jean‐Philippe Azulay (Assistance Publique Hapitaux De Marseille, Marseille, France), Ernest Balaguer Martinez (Hospital General de Catalunya, Barcelona, Spain), Jee Bang (The Johns Hopkins University, Baltimore, MD, USA), Paolo Barone (University of Salerno, Salerno, Italy), Matthew Barrett (University of Virginia Health System, Charlottesville, VA, USA), Danny Bega (Northwestern University, Chicago, IL, USA), Daniela Berg (UKSH ‐ Campus Kiel, Kiel, Germany), Koldo Berganzo Corrales (Hospital De Cruces, Barakaldo, Spain), Yvette Bordelon (University of California, Los Angeles, CA, USA), Adam L Boxer (Memory and Aging Center, Department of Neurology, University of California, San Francisco, CA, USA), Moritz Brandt (Universitatsklinikum Carl Gustav Carus Dresden, Dresden, Germany), Norbert Brueggemann (University Hospital Schleswig‐Holstein, Luebeck, Germany), Giovanni Castelnovo (Centre Hospitalier Universitaire de Nimes ‐ Hopital Universitaire Caremeau, Nimes, France), Roberto Ceravolo (University Hospital of Pisa, Pisa, Italy), Rosalind Chuang (Swedish Health Services, Seattle, WA, USA), Sun Ju Chung (Asan Medical Center, Seoul, Republic of Korea), Alistair Church (Aneurin Bevan University Health Board‐ Clinical Research and Innovation Centre ‐ St Woolos Hospital, Newport, UK), Jean‐Christophe Corvol (Sorbonne Université, Assistance Publique Hôpitaux de Paris, INSERM, CNRS, Institut du Cerveau ‐ Paris Brain Institute ‐ ICM, Hôpital Pitié‐Salpêtrière, Paris, France), Paola Cudia (Biogen, Cambridge, MA, USA), Marian Dale (Medical University of South Carolina, Charleston, SC, USA), Luc Defebvre (Centre Hospitalier Regional Universitaire) de Lille ‐ Hopital Roger Salengro, Lille, France), Sophie Drapier (CHU de Rennes ‐ Hopital de Pontchaillou, Rennes, France), Erika D Driver‐Dunckley (Mayo Clinic Arizona ‐ Scottsdale, Scottsdale, AZ, USA), Georg Ebersbach (Movement Disorders Clinic, Beelitz‐Heilstatten, Germany), Karla M Eggert (Philipps Universitat Marburg, Marburg, Germany), Aaron Ellenbogen (QUEST Research Institute, Farmington Hills, MI, USA), Alexandre Eusebio (Assistance Publique Hapitaux De Marseille, Marseille, France), Andrew H Evans (The Royal Melbourne Hospital (RMH)‐Flemington Neurology ‐ North Melbourne, North Melbourne, Australia), Natalia Fedorova (Russian Medical Academy of Postgraduate Education, Moscow, Russia), Elizabeth Finger (Parkwood Institute, London, Ontario, Canada), Alexandra Foubert‐Samier (CHU De Bordeaux Parkinson Expert Centre, IMNC Hopital Pellegrin, Bordeaux, France), Boyd Ghosh (University Hospital Southampton NHS Foundation Trust, Southampton UK), Lawrence Golbe (Rutgers Robert Wood Johnson Medical School, New Brunswick, NJ, USA), Francisco Grandas Perez (Hospital General Universitario Gregorio Maranon, Madrid, Spain), Murray Grossman (Hospital of the University of Pennsylvania, Philadelphia, PA, USA), Deborah Hall (Rush University Medical Centre Chicago, IL USA), Kyoko Hamada (Shinsapporo Neurosurgical Hospital, Sapporo, Japan), Kazuko Hasegawa (National Hospital Organization Sagamihara National Hospital, Sagamihara, Japan), Guenter Hoeglinger (University Hospital, LMU Munich, Munich, Germany), Lawrence Honig (Columbia University College of Physicians and Surgeons ‐ Gertrude H. Sergievsky Center, New York, NY, USA), David Houghton (Ochsner Medical Center, New Orleans, LA, USA), Xuemei Huang (Penn State University‐Milton S. Hershey Medical Center, Hershey, PA USA), Stuart Isaacson (Parkinson's Disease And Movement Disorder Center Of Boca Raton, Boca Raton, FL, USA), Seong‐ Beom Koh (Korea University Guro Hospital, Seoul, Republic of Korea), Jaime Kulisevsky Bojarski (Hospital de la Santa Creu i Sant Pau, Barcelona, Spain), Anthony E. Lang (Edmond J. Safra Program in Parkinson's Disease and the Rossy PSP Centre, Toronto Western Hospital and the University of Toronto, Toronto, Ontario, Canada), Peter Nigel Leigh (Brighton and Sussex Medical School Trafford Centre for Biomedical Research, Brighton, UK), Irene Litvan (University of California, Parkinson and Other Movement Disorders Center, San Diego, CA, USA), Juan Jose Lopez Lozano (Clinica Ruber Internacional, Madrid, Spain), Jose Luis Lopez‐Sendon Moreno (Hospital Universitario Ramon y Cajal, Madrid, Spain), Albert Christian Ludolph (Universitats‐ und Rehabilitationskliniken Ulm, Ulm, Germany), Ma Rosario Luquin Piudo (Clinica Universidad De Navarra, Pamplona, Spain), Irene Martinez Torres (Hospital la FE, Valencia, Spain), Nikolaus McFarland (University of Florida Center For Movement Disorders and Neurorestoration, Gainesville, FL, USA), Wassilios Meissner (CHU De Bordeaux Parkinson Expert Centre, IMNC Hopital Pellegrin, Bordeaux, France), Tiago Mestre (The Ottawa Hospital ‐ Civic Campus, University of Ottawa, Ottawa, Canada), Pablo Mir Rivera (Hospital Universitario Virgen del Rocio, Sevilla, Spain), Eric Molho (Albany Medical College, Albany, NY, USA), Britt Mollenhauer (Paracelsus‐Elena‐Klinik Kassel, Kassel, Germany), Huw R Morris (National Hospital for Neurology and Neurosurgery, London, UK), Miho Murata (Musashi Hospital, Kodaira‐Shi, Japan), Tomokazu Obi (National Hospital Organization ‐ Shizuoka Institute of Epilepsy and Neurological Disorders, Shizuoka, Japan), Fabienne Ory Magne (Hopital Purpan ‐ Batiment Pierre Paul Riquet, Toulouse, France), Padraig O'Suilleabhain (University of Texas Southwestern Medical Center ‐ Clinical Center For Movement Disorders, Dallas, TX, USA), Rajesh Pahwa (The University of Kansas Medical Center ‐ Parkinson's Disease and Movement Disorder Center, Kansas City, KS, USA), Alexander Pantelyat (The Johns Hopkins University, Baltimore, MD, USA), Nicola Pavese (The Newcastle upon Tyne Hospitals NHS Foundation Trust ‐ Campus for Ageing and Vitality, Newcastle upon Tyne, UK), Dmitry Pokhabov (Federal State Budgetary Institution, Federal Siberian Scientific Clinical Center of Federal Medical‐Biological Agency, Krasnoyarsk, Russia), Johannes Prudlo (Universitaet Rostock ‐ Universitaetsmedizin Rostock, Rostock, Germany), Federico Rodriguez‐Porcel (Medical University of South Carolina, Charleston, SC, USA), James Rowe (Cambridge University, Cambridge, UK), Joseph Savitt (University of Maryland School of Medicine, Baltimore, MD, USA), Alfons Schnitzler (Center for Movement Disorders and Neuromodulation‐University Hospital Dusseldorf, Dusseldorf, Germany), Joerg B Schulz (Uniklinik RWTH Aachen Medizinische Klinik III, Aachen, Germany), Klaus Seppi (Medizinische Universitaet Innsbruck, Innsbruck, Austria), Binit Shah (University of Virginia Health System, Charlottesville, VA, USA), Holly Shill (St. Joseph's Hospital and Medical Center/Barrow Neurology Clinics, Phoenix, AZ, USA), David Shprecher (Banner Sun Health Research Institute, Sun City, AZ, USA), Maria Stamelou (Hygeia Hospital, Marousi, Greece), Malcolm Steiger (The Walton Center ‐ NHS Foundation Trust, Liverpool, UK), Yuji Takahashi (Musashi Hospital, Kodaira‐Shi, Japan), Hiroshi Takigawa (Tottori University Hospital, Yonago, Japan), Carmela Tartaglia (Toronto Western Hospital, University Health Network Movement Disorders Centre, Toronto, Canada), Lars Toenges (St. Josef ‐ Hospital Bochum, Kardiologische Studienambulanz, Bochum, Germany), Daniel Truong (The Parkinson's and Movement Disorder Institute, Fountain Valley, CA, USA), Winona Tse (Mount Sinai Movement Disorders Center, New York, NY, USA), Paul Tuite (University of Minnesota Medical Center ‐ Fairview ‐ Neurology Clinic, Minneapolis, MN, USA), Dieter Volc (Prosenex Ambulatoriumsbetriebs GmbH – Studienzentrum, Vienna, Austria), Anne‐Marie A Wills (Massachusetts General Hospital Cancer Center, Boston, MA, USA), Dirk Woitalla (St. Josef‐ Krankenhaus, Essen‐Kupferdreh, Essen, Germany), Tao Xie (The University of Chicago Medicine ‐ Center for Parkinson's Disease and Movement Disorders, Chicago, IL, USA), Tatsuhiko Yuasa (Kamagaya General Hospital, Kamagaya‐City, Japan), Sarah Elizabeth Zauber (Indiana University Health Physicians ‐ Neurology – Indianapolis, Indianapolis, IN, USA), Theresa Zesiewicz (University of South Florida ‐ Morsani College of Medicine, Tampa, FL, USA).

## A.2 Alzheimer's Disease Neuroimaging Initiative (ADNI)

A complete list of ADNI investigators can be found at: http://adni.loni.usc.edu/wp-content/uploads/how_to_apply/ADNI_Acknowledgement_List.pdf.

## A.3 The AL‐108‐231 Study Group

Australia: David Williams.

Canada: Anne Louise Lafontaine, Connie Marras, Mandar Jog, Michael Panisset, Anthony Lang, Lesley Parker, Alistair J. Stewart.

France: Jean‐Christophe Corvol, Jean‐Philippe Azulay, Philippe Couratier.

Germany: Brit Mollenhauer, Stefan Lorenzl, Albert Ludolph, Reiner Benecke, Günter Höglinger, Axel Lipp, Heinz Reichmann, Dirk Woitalla.

United Kingdom: Dennis Chan, Adam Zermansky, David Burn, Andrew Lees.

United States: Adam Boxer, Bruce L. Miller, Iryna V. Lobach, (Memory and Aging Center, Department of Neurology, University of California, San Francisco, CA, USA), Erik Roberson, Lawrence Honig, Edward Zamrini, Rajesh Pahwa, Yvette Bor‐delon, Erika Driver‐Dunkley, Stephanie Lessig, Mark Lew, Kyle Womack, Brad Boeve, Joseph Ferrara, Argyle Hillis, Daniel Kaufer, Rajeev Kumar, Tao Xie, Steven Gunzler, Theresa Zesiewicz, Praveen Dayalu, Lawrence Golbe, Murray Grossman, Joseph Jancovic, Scott McGinnis, Anthony Santiago, Paul Tuite, Stuart Isaacson, Julie Leegwater‐Kim, Irene Litvan, Murray Grossman, David S. Knopman, Bruce L. Miller, Lon S. Schneider, Rachelle S. Doody, Lawrence I. Golbe, Erik D. Roberson, Mary Koestler, Clifford R. Jack Jr., Viviana Van Deerlin, Christopher Randolph, Iryna V. Lobach, Illana Gozes, Steve Whitaker, Joe Hirman, Michael Gold, Bruce H. Morimoto.
