# Supplementary figures and images for: Brain Networks Route Neurodegeneration Patterns in Patients with Progressive Supranuclear Palsy
Source: Mov Disord. 2025 Jun 9;40(10):2102–15. doi: 10.1002/mds.30257 (PMC12553996; doi:10.1002/mds.30257)

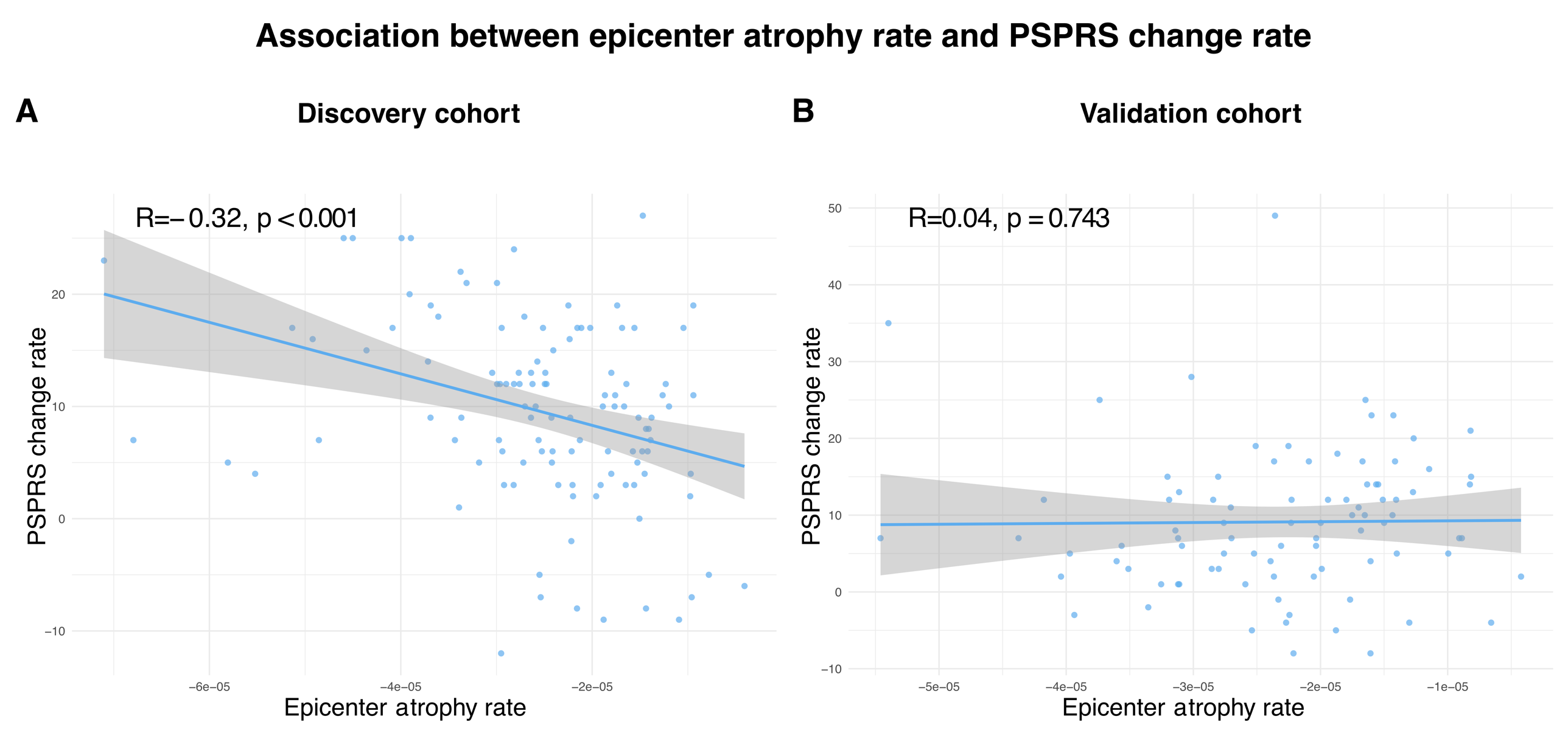

Supplement: Supplementary file 1 — Figure S1. Association between epicenter atrophy rate and PSPRS change rate. [file MDS-40-2102-s002.jpg]
