# Supplementary material for: Brain Networks Route Neurodegeneration Patterns in Patients with Progressive Supranuclear Palsy
Source: Mov Disord. 2025 Jun 9;40(10):2102–15. doi: 10.1002/mds.30257 (PMC12553996; doi:10.1002/mds.30257)
Supplement: Supplementary file 2 — Data S1. Supporting Information. [file MDS-40-2102-s001.docx]

**Supplementary Methods**

***Sample***

For our primary analyses, we included two independent multisite cohorts of patients with PSP-RS from the placebo arms of two negative randomized controlled trials (PASSPORT [NCT03068468]^1^, and AL-108-231 Davunetide [NCT01110720]^2^), which examined patients with PSP-RS with similar study protocols including longitudinal MRI assessments spanning 52 weeks. We included patients from the PASSPORT [NCT03068468] trial (n=114) as the primary discovery cohort, while patients from AL-108-231 Davunetide trial [NCT01110720] (n=90) were used as an independent validation cohort. For the current study, we specifically selected patients which met diagnosis of probable PSP-RS according to the current MDS PSP criteria^3^; to ensure highest likelihood of underlying 4R tau pathology. All subjects had available demographic data as well as baseline and 52-week-follow-up 3D T1-weighted MRI data. Detailed information and inclusion criteria can be found in previous publications ^4^ and online (https://clinicaltrials.gov) with the respective trial identifiers. For replication and the assessment of robustness, all analyses were determined independently in both the discovery and validation PSP-RS patient samples.

As a healthy reference cohort to determine regional brain atrophy patterns in the PSP-RS cohorts, we further included 3T structural MRI data from n=377 cognitively normal (i.e., Mini Mental State Examination [MMSE]≥24, Clinical Dementia Rating [CDR]=0, non-depressed) Florbetaben amyloid-PET and Florbetaben tau-PET negative individuals from the Alzheimer’s Disease Neuroimaging Initiative (ADNI, ClinicalTrials.gov ID: NCT02854033), who underwent a highly congruent structural MRI protocol as the PSP-RS cohorts. We specifically selected amyloid-PET and tau-PET negative subjects ADNI to rule out any confounding Alzheimer’s neurodegenerative pathology that may drive MRI-assessed neurodegeneration. Cut-offs to determine negativity on PET biomarkers in the ADNI sample were based on pre-established thresholds, i.e., a global SUVR below 1.11 for Florbetapir amyloid-PET using a whole cerebellum reference^5^ and a global SUVR below 1.3 for Flortaucipir tau-PET using an inferior cerebellar grey reference^6^. For assessing brain connectivity, we further included rs-fMRI data from a selected subset of n=69 of amyloid- and tau-PET negative cognitively normal ADNI subjects who underwent 3T rs-fMRI obtained with a harmonized protocol within six months of the amyloid- and tau-PET assessments.

***Structural MRI acquisition and assessment of grey matter atrophy***

All structural MRI data in the two PSP-RS cohorts as well as the ADNI control sample was obtained with a harmonized protocol that followed the ADNI recommendations for volumetric MRI analysis. Specifically, all subjects underwent 3D T1-weighted 3D magnetization prepared rapid gradient echo (MPRAGE) imaging with a 1x1x1mm resolution and a TR of 2300ms. All scans were inspected for artifacts prior and after segmentation to ensure sufficient quality of the included MRI datasets. All subject-level structural MRI scans were processed using the CAT12 toolbox for volumetric analyses (https://neuro-jena.github.io/cat/) and were bias-corrected, segmented into grey matter, white matter, and cerebrospinal fluid segments, Jacobian modulated and non-linearly warped to Montreal Neurological Institute (MNI) space using the CAT12 toolbox. For the two longitudinal PSP-RS patient cohorts, we specifically selected processing of longitudinal MRI data, which includes an initial generation of a subject-level MRI template followed by joint spatial normalization, which aids in the detection of within-subject volume changes over time. For each subject and time-point, we applied the Brainnetome atlas^7^ to Jacobian modulated and MNI normalized grey matter segments, to obtain grey matter volume per region of interest (ROI). All ROI level grey matter volumes were adjusted to the total intracranial volume to adjust for differences in head size between individuals. To determine abnormality in grey matter volume, we used w-scores, i.e., a modification of z-scores that define normality or abnormality while adjusting for covariates that may confound grey matter volume changes such as age and sex.^8^ To assess w-scores of grey matter volume, we first determined the effect of age and sex on grey matter volume changes using the 377 ADNI cognitively normal controls as a reference. Specifically, we determined regression models for each Brainnetome ROI with age and sex as a predictor of grey matter volume. These regression models were subsequently applied to the PSP-RS patient data, to estimate the expected grey matter volume per ROI and subject given a subjects’ age and sex. Subsequently, we calculated the difference between a given PSP-RS subjects’ actual grey matter volume and the volume estimated purely based on age and sex, which was subsequently divided by the standard deviation of the grey matter volume in the ADNI cognitively normal controls. The resulting w-scores therefore indicate the difference between a subject’s actual grey matter volume relative to what is expected by their age and sex, relative to the standard deviations of grey matter volume in controls. These w-scores can be interpreted as z-scores, where negative values indicate stronger atrophy than expected merely by age and sex.

For longitudinal grey matter volume changes, we determined subject level volume changes per ROI adjusted for total intracranial volume, rather than using w-scores, given that each individual defines its own baseline volume allowing to infer the directionality of changes. To estimate grey matter volume changes, annual change rates were defined for each ROI as the difference in MRI-assessed grey matter volume between the follow-up and baseline visit divided by the time between the visits (52 weeks).

***Assessment of a functional connectivity template***

3T rs-fMRI from 69 ADNI healthy controls was used to determine a connectivity template across which we modelled spread of grey matter atrophy as described previously^9^. To determine a functional connectivity template for modelling the expansion of grey matter atrophy, we included rs-fMRI data from 69 out of the 377 cognitively normal amyloid-PET and tau-PET negative ADNI controls, who had available 10-minute rs-fMRI (i.e., echo-planar imaging [EPI]) data that was consistently acquired on 3T Siemens scanners with a uniform rs-fMRI protocol (TR/TE=3000ms/90ms). Rs-fMRI images were slice-time and motion corrected and co-registered to their respective T1-weighted images. Using rigid-transformation parameters, T1-derived grey-matter, eroded white matter and eroded cerebrospinal fluid (CSF) segments were transformed to EPI space. To denoise EPI images, we regressed out nuisance covariates (i.e., eroded white matter and eroded CSF timeseries plus six motion parameters) and applied detrending and band-pass filtering (0.01-0.08Hz) in EPI native space. To further reduce movement artifacts which may compromise connectivity assessment^10^, we performed motion scrubbing in which volumes exceeding a 0.5mm frame-wise displacement threshold were removed, as well as one prior and two subsequent volumes. All subjects had at least five minutes of rs-fMRI remaining after scrubbing^11^. Spatial smoothing was not carried out to avoid artificially enhancing functional connectivity caused by signal spilling between adjacent brain regions. Pre-processed rs-fMRI images were subsequently warped to MNI space using the CAT12-derived spatial normalization parameters. Subject-specific functional connectivity matrices were determined across the 246 ROIs of the Brainnetome atlas as Fisher-z-transformed Pearson moment correlations between ROI-specific time-series. All individual matrices were averaged and thresholded at 30% density, with negative connections excluded, following our previously established protocol to maximize consistency across studies ^12,13^. The average functional connectivity was then converted to a distance-based connectivity matrix ^14^, where shorter path-lengths between ROIs represent stronger connectivity, in line with our previous work^13,15^.

***Association with clinical status***

We determined the association between epicenter atrophy rate (i.e., regions with fastest volume decline over time, defined as the top 10% of ROIs with highest atrophy) and PSP-rating scale (PSPRS) change rate, calculating a partial correlation adjusted for age and sex. The PSPRS change rates were calculated as the difference between scores of PSPRS_follow-up_ and PSPRS_baseline_. Positive PSPRS change rates indicate clinical worsening over time while negative PSPRS change rates indicate improvement.

**Supplementary Figure**

**Supplementary Figure 1: Association between epicenter atrophy rate and PSPRS change rate**

Partial correlation (adjusted for age and sex) between epicenter atrophy rate (defined as the top 10% of ROIs with the highest atrophy, i.e. the regions with fastest volume decline over time) and PSP-rating scale (PSPRS) change rate (scores PSPRS_follow-up_ − PSPRS_baseline_) is shown for the Discovery cohort (A) and the Validation cohort (B). Positive PSPRS-values indicate clinical worsening over time; negative PSPRS-values indicate improvement.

**Supplementary references**

1. Dam T, Boxer AL, Golbe LI, et al. Safety and efficacy of anti-tau monoclonal antibody gosuranemab in progressive supranuclear palsy: a phase 2, randomized, placebo-controlled trial. *Nat Med*. 2021;27(8):1451-1457. doi:10.1038/s41591-021-01455-x

2. Boxer AL, Lang AE, Grossman M, et al. Davunetide in patients with progressive supranuclear palsy: a randomised, double-blind, placebo-controlled phase 2/3 trial. *Lancet Neurol*. 2014;13(7):676-685. doi:10.1016/S1474-4422(14)70088-2

3. Höglinger GU, Respondek G, Stamelou M, et al. Clinical diagnosis of progressive supranuclear palsy: The movement disorder society criteria. *Mov Disord Off J Mov Disord Soc*. 2017;32(6):853-864. doi:10.1002/mds.26987

4. Quattrone A, Franzmeier N, Huppertz HJ, et al. Magnetic Resonance Imaging Measures to Track Atrophy Progression in Progressive Supranuclear Palsy in Clinical Trials. *Mov Disord*. 2024;39(8):1329-1342. doi:10.1002/mds.29866

5. Landau SM, Mintun MA, Joshi AD, et al. Amyloid Deposition, Hypometabolism, and Longitudinal Cognitive Decline. *Ann Neurol*. 2012;72(4):578-586. doi:10.1002/ana.23650

6. Maass A, Landau S, Baker SL, et al. Comparison of multiple tau-PET measures as biomarkers in aging and Alzheimer’s disease. *NeuroImage*. 2017;157:448-463. doi:10.1016/j.neuroimage.2017.05.058

7. Fan L, Li H, Zhuo J, et al. The Human Brainnetome Atlas: A New Brain Atlas Based on Connectional Architecture. *Cereb Cortex N Y N 1991*. 2016;26(8):3508-3526. doi:10.1093/cercor/bhw157

8. La Joie R, Perrotin A, Barré L, et al. Region-specific hierarchy between atrophy, hypometabolism, and β-amyloid (Aβ) load in Alzheimer’s disease dementia. *J Neurosci Off J Soc Neurosci*. 2012;32(46):16265-16273. doi:10.1523/JNEUROSCI.2170-12.2012

9. Franzmeier N, Brendel M, Beyer L, et al. Tau deposition patterns are associated with functional connectivity in primary tauopathies. *Nat Commun*. 2022;13(1):1362. doi:10.1038/s41467-022-28896-3

10. Power JD, Mitra A, Laumann TO, Snyder AZ, Schlaggar BL, Petersen SE. Methods to detect, characterize, and remove motion artifact in resting state fMRI. *NeuroImage*. 2014;84:320-341. doi:10.1016/j.neuroimage.2013.08.048

11. Franzmeier N, Düzel E, Jessen F, et al. Left frontal hub connectivity delays cognitive impairment in autosomal-dominant and sporadic Alzheimer’s disease. *Brain J Neurol*. 2018;141(4):1186-1200. doi:10.1093/brain/awy008

12. Frontzkowski L, Ewers M, Brendel M, et al. Earlier Alzheimer’s disease onset is associated with tau pathology in brain hub regions and facilitated tau spreading. *Nat Commun*. 2022;13:4899. doi:10.1038/s41467-022-32592-7

13. Franzmeier N, Dewenter A, Frontzkowski L, et al. Patient-centered connectivity-based prediction of tau pathology spread in Alzheimer’s disease. *Sci Adv*. 2020;6(48):eabd1327. doi:10.1126/sciadv.abd1327

14. Rubinov M, Sporns O. Complex network measures of brain connectivity: Uses and interpretations. *NeuroImage*. 2010;52(3):1059-1069. doi:10.1016/j.neuroimage.2009.10.003

15. Pichet Binette A, Franzmeier N, Spotorno N, et al. Amyloid-associated increases in soluble tau relate to tau aggregation rates and cognitive decline in early Alzheimer’s disease. *Nat Commun*. 2022;13:6635. doi:10.1038/s41467-022-34129-4
